# DNAJB6-Containing Extracellular Vesicles as Chaperone Delivery Systems: A Proteomic Analysis

**DOI:** 10.3390/pharmaceutics14112485

**Published:** 2022-11-17

**Authors:** Bhagyashree S. Joshi, Hector Garcia Romeu, Aldy Aliyandi, Marcel P. de Vries, Inge S. Zuhorn

**Affiliations:** 1 Department of Biomedical Engineering, University Medical Center Groningen, University of Groningen, Antonius Deusinglaan 1, 9713AV Groningen, The Netherlands; 2 Groningen Research Institute of Pharmacy, University Medical Center Groningen, University of Groningen, Antonius Deusinglaan 1, 9713AV Groningen, The Netherlands; 3 Interfaculty Mass Spectrometry Center, University Medical Center Groningen, University of Groningen, Antonius Deusinglaan 1, 9713AV Groningen, The Netherlands

**Keywords:** extracellular vesicles, exosomes, chaperones, DNAJB6, proteome, drug delivery

## Abstract

Cell-derived extracellular vesicles (EVs) are effectors of cell-to-cell communication that are in the spotlight as promising candidates for in vivo drug delivery because of their ability to enter cells and deliver cargo. For example, proteins of interest can be loaded into EVs to mediate protein transfer into target cells. To determine causality between EV content and function, which is also important to assess the clinical safety of EVs, it is crucial to comprehensively characterize their complete molecular composition. Here, we investigated EVs loaded with the chaperone protein DNAJB6. Chaperone proteins assist in protein folding and have been suggested to alleviate protein aggregation diseases, such as Alzheimer’s disease and Huntington’s disease. We analyzed and compared the proteome of EVs isolated from wildtype HEK293T cells with that of EVs from HEK 293T cells overexpressing DNAJB6-WT or loss-of-function mutant DNAJB6-M3. Comprehensive analysis of proteomics data showed enhanced levels of DNAJB6 as well as protein-folding-related proteins in EVs derived from DNAJB6-overexpression cells. Interestingly, upregulation of a chaperone and its protein-folding-related proteins resulted in downregulation of another chaperone plus its related proteins, and vice versa. This implies the presence of compensatory mechanisms in the cellular expression of chaperones. Collectively, we provide the proteomic EV signatures underlying EV mediated DNAJB6 transmission by HEK293T cells, with the aim of establishing a causal relationship between EV protein content and EV function.

## 1. Introduction

Extracellular vesicles (EVs) are membrane-enclosed nanoparticles with sizes ranging from 50 nm to a few μm, which are mediators of intercellular communication [1,2]. So-called producer (or donor) cells release EVs into the extracellular environment, where they can bind and become internalized by acceptor (or recipient) cells. EVs play an important role in a myriad of biological processes under both normal and diseased conditions [3]. Depending upon the producer cell type and state, EVs contain a specific combination of functional biomolecules, including structural proteins, signaling proteins, lipids, and nucleic acids [4]. Several studies have reported functional delivery of these biomolecules to target cells, highlighting their role as natural delivery vehicles [5,6,7,8,9,10,11]. Importantly, due to their low immunogenicity, high specificity, and delivery efficiency compared to traditional delivery vehicles (liposomes, viruses, polymeric nanoparticles, etc.) [12,13], EVs offer significant promise as drug delivery systems.

Recently, it was shown that cells overexpressing HSP40 and HSP70, chaperones involved in protein folding, could restore protein homeostasis in neighboring cells in murine and drosophila brain [14]. EVs secreted from HSP40- or HSP70-overexpressing cells carried the chaperones and were taken up by other cells, resulting in a reduction in protein aggregation in these recipient cells. Similarly, astrocytic overexpression of DNAJB6, a potent suppressor of polyglutamine-mediated protein aggregation, was shown to reduce protein aggregation in neurons in drosophila brain [15]. Although the exact mechanism was not deciphered, it was speculated that this effect could be attributed to EV-mediated transfer of chaperones to neighboring brain cells. Thus, chaperone-rich EVs may promote proteostasis in recipient cells. Such EV-mediated transfer of chaperones may prove to be useful for treatment of neurodegenerative diseases, where the primary cause of pathogenesis is severe protein misfolding [16]. However, realization of such clinical potency warrants a detailed description of EV composition to identify potential harmful and/or synergistic components, which is important to assess the potential of the use of EVs for drug delivery applications. 

Proteomics is a very effective way to discern the global proteome of (engineered) EVs [17]. First, it is useful to validate the presence of the cargo protein of interest in EVs. Further, it reveals the entire set of proteins present in the EVs, which may also exert a function in target cells. Collectively, such comprehensive analysis will provide information about all proteins that are delivered to recipient cells via EVs and the cellular pathways in which they are involved and, thus, may affect [18,19]. 

In the present study, we analyzed the protein contents of EVs isolated from cells overexpressing the wildtype and mutant forms of the chaperone DNAJB6 by label-free liquid chromatography−tandem mass spectrometry (LC−MS/MS). A total of 1806, 1830, and 1807 proteins were identified in EVs from HEK293T Wildtype (WT), HEK293T-DNAJB-WT overexpression (DNAJB6-WT), and DNAJB6-M3 overexpression (DNAJB6-M3) cell lines, respectively. Overexpression of DNAJB6-WT and DNAJB6-M3 resulted in differential abundance (greater than two-fold) of 166 and 191 proteins with respect to control WT EVs, respectively. Among the enriched proteins were DNAJA1, HSPB1, DNAJB2 chaperones, ribosome proteins, and histones, while mitochondrial proteins were highly downregulated compared to WT EVs, suggesting that EVs isolated from DNAJB6 overexpression seem beneficial for use as delivery vehicles of chaperones and other proteins involved in protein folding. In addition, the chaperone content of the EVs enlarges with not only DNAJB6 but also other chaperone proteins, thus widening their applicability to other diseases provided the function of accompanying chaperones is proven to be beneficial. 

## 2. Materials and Methods

### 2.1. Plasmids

GFP-DNAJB6b-WT and GFP-DNAJB6b-M3 DNA sequences were amplified from plasmids containing pcDNA5/FRT/TO GFP-DNAJB6b-WT and pcDNA5/FRT/TO GFP-DNAJB6b-M326 (a kind gift from Prof. Harrie H. Kampinga), adding BsmBI site at both the ends of the sequence. The amplified segments were inserted into an entry vector pENTR1A (a gift from Eric Campeau and Paul Kaufman; Addgene, plasmid# 17398; PRID:Addgene_17398) by golden gate assembly. The resultant plasmids were recombined with pLenti-CMV-Puro-DEST (a gift from Eric Campeau and Paul Kaufman; Addgene, plasmid# 17452; PRID:Addgene_17452) using a gateway LR clonase enzyme (ThermoFisher Scientific, 11791100) to achieve the expression vector pLenti-CMV-GFP-DNAJB6-WT-Puro-DEST and pLenti-CMV-GFP-DNAJB6-M3-Puro-DEST. pEGFP-Q74 was a gift from David Rubinsztein (Addgene plasmid #40262; PRID:Addgene_40262). Full sequences of used proteins are provided in the Supplementary Materials. Amino acid residues changed in M3 with respect to WT DNAJB6 sequence are marked yellow.

### 2.2. Antibodies and Reagents

Primary antibodies against GFP (mouse; Clontech 632381; 1:5000), DNAJB6 (rabbit; housemade; 1:2000), TSG101 (rabbit; Abcam ab92726; 1:1000), and Calnexin (rabbit; StressGen ADI-SPA-860; 1:2000) were used. Odyssey secondary antibodies—anti-mouse and -rabbit antibodies (Li-COR, LI 926-68070, LI 926-32211, and LI 926-32219) were used at 1:5000 dilution. 

### 2.3. Preparation of EV-Depleted Medium

DMEM (10% FBS, 1% Pen-Strep) was centrifuged at 110,000× *g* at 4 °C for 16 h to remove the fetal-bovine-serum-derived EVs. The supernatant was collected and sterilized by filtration through a 0.2 μm filter (Millipore, Merck Life Science, Amsterdam, The Netherlands) and stored at 4 °C until further use.

### 2.4. Cell Culture 

HEK293T cells were cultured in DMEM (Gibco, 41965-039, ThermoFisher, Landsmeer, The Netherlands) supplemented with 10% fetal bovine serum (FBS, Bodinco, 5010, Alkmaar, The Netherlands) and 1% Penicillin–Streptomycin sulfate (Gibco, 15140-122) at 37 °C under 5% CO_2_. 

### 2.5. Co-Culture of DNAJB6-WT and DNAJB6-M3 Overexpression Cells with HEK293T-Q74-GFP Cells, and Microscopy Analysis of PolyQ Aggregates

2 × 10^5^ HEK293T cells in 0.5 mL medium were plated on 6-well plate transwell filters (Millipore, PIHP03050) 1 day prior to transfection. 2 mL was added in the well beneath the transwell filters. The next day, the cells were transfected with mock, DNAJB6-WT, and DNAJB6-M3 plasmids using Lipofectamine 2000 (ThermoFisher, 11668027, Landsmeer, The Netherlands) and incubated with lipoplexes for 4 h at 37 °C in serum-free medium. The medium beneath the filter was also changed to serum-free medium. All the medium was refreshed with EV-depleted medium and cells were incubated further for 24 h at 37 °C.

In another dish, 3 × 10^5^ HEK293T cells were seeded on a glass cover slip at cell density of 150,000 cells/mL in 2 mL and incubated overnight at 37 °C in a 6-well plate. The next day, the cells were transfected with 1 μg Q74-GFP encoding plasmid in 1 ml serum-free medium per transwell for 4 h with Fugene HD (Promega, E2311, Leiden, The Netherlands), after which cells were washed with HBSS. The medium cultured beneath the transwells was added to these cells to replace HBSS. The transwell filters with mock-, DNAJB6-WT-, and DNAJB6-M3-transfected cells along with the medium already containing EVs were then placed in the wells containing HEK293T-Q74-GFP cells and co-incubated for 20 h. Next, the HEK293T-Q74-GFP cells were subjected to microscopic analysis using a fluorescence microscope (Leica DM6000, Wetzlar, Germany; for a more detailed protocol of microscopic analysis, see our STAR protocol [20]). Five random image fields were selected per sample and polyQ aggregates and nuclei were counted for each image. The aggregates/nuclei ratio was calculated for each sample. Data are shown as the mean ± SEM of three independent experiments.

### 2.6. EV Production

For isolation of EVs, 5–8 × 10^6^ HEK293T cells were seeded in a 15 cm culture dish with complete media. The next day, cells were transfected using Lipofectamine 2000 (Invitrogen, 11668027, ThermoFisher, Landsmeer, The Netherlands) with 15 μg pcDNA5/FRT/TO GFP-DNAJB6b-WT or pcDNA5/FRT/TO GFP-DNAJB6b-M3 plasmid/dish for 4 h at 37 °C in 10 mL serum-free medium. For control EVs, cells were transfected with pcDNA5/FRT/TO empty (mock) plasmid, keeping the rest of the procedures unchanged. Post transfection, the cells were washed with Hanks Balanced Salt Solution (HBSS, Gibco, 14025092), and EV-depleted medium was added. Culture media were collected after 48 h, followed by EV isolation (at cell confluency of 90–95%; see Section 2.8). 

Separately, on a 13 mm glass coverslip/well in a 24-well plate, 5 × 10^4^ HEK293T WT cells were seeded in complete medium. The next day, cells were washed with pre-warmed HBSS and 100 μg/mL EVs isolated from GFP-DNAJB6-WT overexpression cells were added in 200 μL EV-depleted medium and incubated for 24 h. 

### 2.7. Stable Cell Line Generation and EV Production for LC-MS/MS Experiments

HEK293T-GFP-DNAJB6b-WT and HEK293T-GFP-DNAJB6b-M3 stable cell lines were generated via lentiviral transduction of HEK293T followed by Puromycin (Sigma P8833, Merck Life Science, Amsterdam, The Netherlands) selection at 1 µg/mL until no further cell death was observed. Thereafter, cells were cultured in complete medium without antibiotic.

10–12 × 10^6^ cells of each cell line (HEK293T-WT, HEK293T-GFP-DNAJB6b-WT, and HEK293T-GFP-DNAJB6b-M3) were seeded/flask in EV-depleted medium (three T162 flasks per condition), and EVs were isolated after 48 h (at cell confluency of 90–95%; see Section 2.8). 

### 2.8. EV Isolation and Characterization

EVs were isolated from conditioned media by sequential centrifugation. First, dead cells were removed from the supernatant by centrifugation at 500× *g* for 10 min, followed by centrifugation at 2000× *g* for 10 min to remove cellular debris (Beckman Coulter, Allegra X-15R). Centrifugation of the resultant supernatant at 10,000 g for 30 min (Sorvall Discovery 90SE ultracentrifuge using Beckman SW32i rotor) removed apoptotic vesicles and microvesicles. Finally, the resultant supernatant was subjected to ultracentrifugation at 110,000× *g* for 70 min to collect EVs as a pellet and washed with 5 mL of PBS and pelleted again by ultracentrifugation at the same conditions using Beckman SW55i rotor. The final EV pellet was resuspended in 50 µL PBS and stored at 4 °C until further use.

### 2.9. Protein Quantification and Western Blotting

Protein concentration of the EV solution was measured with DC protein assay kit (Bio-Rad, 5000114, Lunteren, The Netherlands). 30 µg of total EV protein or whole cell lysate, after boiling at 95 °C for 5 min with 4X Laemmelli loading buffer, was loaded in separate wells onto an SDS-PAGE gel, run at 80 V for 1.5 h, transferred to a PVDF membrane (Millipore, IPFL00010, Merck Life Science, Amsterdam, The Netherlands), and blocked with Odyssey blocking buffer (Li-COR, 927-40000, Bad Homburg, Germany) for 1 h at RT. Blots were incubated with primary antibodies in blocking buffer overnight at 4 °C and washed with 0.1% PBS-Tween20. Subsequently, blots were probed with secondary antibody solution for 1 h at RT and washed with 0.1% PBS-Tween20. The images were acquired with an Odyssey^®^ Infrared Imaging System (Li-COR, Bad Homburg, Germany).

### 2.10. Sample Preparation for LC-MS/MS 

EVs were isolated from the cell culture media of the three stable cell lines (n = 3). For each cell line, the 3 samples were pooled and subjected to LC-MS/MS. 

For each sample, 30 µg of total EV protein in a total volume of 15 µL was mixed with 0.1% Rapigest (Waters, Milford, MA, USA) in 1:1 (*v/v*) ratio and incubated 10 min at room temperature. Resultant solution was digested with mass spec grade trypsin (Thermoscientific, 90057) at 37 °C for 3 h. Digestion was stopped and RapiGest was hydrolyzed by adding 0.5% Formic Acid (FA) in Milli-Q water. Samples were then desalted on Gracepure^TM^-C18-AQ Reversed-Phase SPE column (Grace DavisonTM 5141523). Resultant eluents were lyophilized and re-suspended in 0.1% FA before MS analysis.

### 2.11. LC-MS/MS Analysis 

Proteomics analyses were performed using an UltiMate 3000 RSLC UHPLC system connected to an Orbitrap Q Exactive Plus mass spectrometer operating in data-dependent acquisition (DDA) mode. A sample volume corresponding to 1 μg of total protein (based on the micro-BCA assay) was injected onto an Acclaim PepMap100 C18 trap column using μLpickup sample loading mode with 0.1% FA in H2O at 20 μL/min. Peptides were separated on a 50 cm Acclaim PepMap RSLC C18 analytical column, which was kept at 40 °C, using a 117 min linear gradient from 3 to 40% eluent B (0.1% FA in ACN) in eluent A (0.1% FA in H_2_O) at a flow rate of 200 nL/min. For DDA, survey scans from 300 to 1650 m/z were acquired at a resolution of 70,000 (at 200 m/z) with an AGC target value of 3 × 10^6^ and a maximum ion injection time of 50 ms. From the survey scan, a maximum number of 12 of the most abundant precursor ions with a charge state of 2+ to 6+ were selected for higher energy collisional dissociation (HCD) fragment analysis between 200 and 2000 m/z at a resolution of 17,500 (at 200 m/z) with an AGC target value of 5 × 10^4^, a maximum ion injection time of 50 ms, a normalized collision energy of 28%, an isolation window of 1.6 m/z, an underfill ratio of 1%, an intensity threshold of 1 × 10^4^, and the dynamic exclusion parameter set at 20 s.

### 2.12. Data Processing 

Shotgun proteomics data were processed using the freely available MaxQuant computational platform [21] (https://www.maxquant.org/; accessed on 5 March 2019) with default parameters, performed in the Interfaculty Mass Spectrometry Center at UMCG. For protein quantification, proteomics data were scaled by median scale normalization. Logarithmic transformation (base 10) of intensity values was applied prior to characterizing differential expression, in which proteins changing at least two-fold in expression were identified.

### 2.13. Gene Ontology (GO) and Pathway Analysis 

All the experimental evidence for WT, DNAJB6-WT, and DNAJB6-M3 EV proteins was used for GO Biological Process, KEGG pathway, and Up_Tissue annotations using The Database for Annotation, Visualization and Integrated Discovery (DAVID) v6.8 [22,23], using default DAVID criteria of significance value of <0.01 and Bonferroni correction method. 

### 2.14. Signal Peptide Analysis Using SignalP-5.0

DNAJB6 (Uniprot ID: O75190) protein sequence was extracted from Uniprot database and entered into the SignalP-5.0 [24] search field. Eukarya and Long output options were chosen before submission of the query sequence.

### 2.15. Venn Diagrams

All diagrams were made with VENNY 2.1 [25]. Protein official gene symbols were used for generating the Venn diagrams.

### 2.16. Statistics

Graphical data were analyzed using GraphPad prism version 7.0 using one-way ANOVA using Tukey’s multiple correlation test. Data are presented as mean ± SD (** *p* <0.01; ns, not significant).

## 3. Results

### 3.1. Elevated DNAJB6b Expression in HEK293T Cells Improves Q74-GFP Protein Folding in Co-Cultured Q74-GFP-Expressing HEK293T Cells

**Figure 1:**
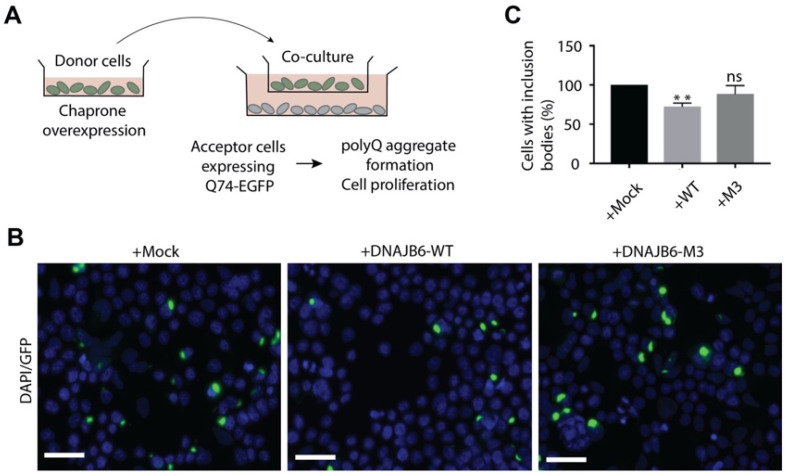
Cell-non-autonomous reduction in polyQ aggregation in Q74-EGFP-expressing HEK293T cells upon co-culture with DNAJB6-WT-expressing HEK293T cells. (**A**) Co-culture assays setup for assessment of overexpression of DNAJB6 on polyQ aggregation in recipient cells. (**B**) Representative fluorescence microscopy images of Q74-GFP expressing recipient cells co-incubated with mock, DNAJB6-WT, or DNAJB6-M3 transfected HEK293T donor cells. Bar: 40 µm. DAPI: nuclei, GFP: polyQ aggregates. (**C**) Graphical representation of microscopy analysis Q74-GFP aggregates resulted from co-culture experiments (n = 3, number of cells counted >900 per condition, mean ± SEM, ** *p* < 0.01, ns: not significant).

Several lines of research have suggested that chaperone-rich EVs may promote proteostasis in recipient cells. To provide evidence for such cell-non-autonomous induction of proteostasis, we set out to investigate whether DNAJB6-overexpressing cells could improve protein folding in recipient cells in the absence of direct cell–cell contact. To this end, cells transfected with mock, GFP-DNAJB6-WT, and GFP-DNAJB6-M3 plasmids were separately seeded on cell-culture inserts and co-incubated with HEK293T cells expressing Q74-GFP, which were grown in the basal chamber of a transwell filter system (Figure 1A). Q74-GFP is an aggregation-prone protein that readily forms insoluble aggregates (inclusion bodies) in cells, which can be easily visualized by fluorescence microscopy. Overexpression of DNAJB6 has been reported to suppress such polyglutamine inclusions, while DNAJB6-M3 is a mutant form of DNAJB6 with loss of aggregation inhibition function as a result of S/T-A substitutions in the S/T rich region [26]. Co-culture of DNAJB6-WT overexpression cells led to a 22 ± 5.3% decrease in the number of cells containing Q74-GFP aggregates in comparison to mock transfected cells, whereas cells overexpressing the non-functional mutant DNAJB6-M3 did not have a significant effect on polyQ aggregation (11.2 ± 11.6% decrease) (Figure 1B,C). Taken together, these results indicate that DNAJB6 overexpression in cells can affect protein folding in co-cultured cells in the absence of direct cell–cell contact, suggesting a role for secreted factors. To check for the possibility of secretion of ‘free’ DNAJB6 from cells, we checked for the presence of a putative secretion signal peptide in the DNAJB6 protein sequence. A very low putative secretion signal peptide sequence likelihood was found for DNAJB6 (Supplementary Figure S1).

Alternatively, the data suggest a possible role for EVs as DNAJB6 transmitters, as was already speculated to be responsible for the protective role of DNAJB6-expressing astrocytes in polyQ-expressing neurons in drosophila [15].

**Figure 2:**
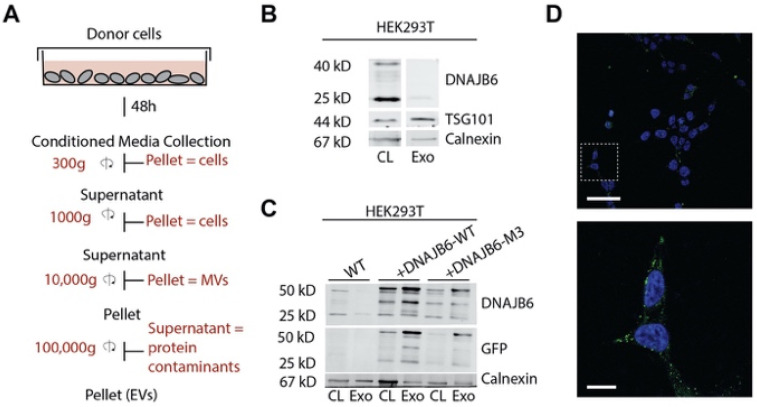
DNAJB6-WT- and DNAJB6-M3-expressing HEK293T cells release exosomes that contain DNAJB6-WT and DNAJB6-M3, respectively. (**A**) Exosome isolation workflow from cell culture media of donor cells. (**B**) Western blot analysis of exosomes isolated from HEK293T cells for DNAJB6, exosome marker TSG101, and non-marker Calnexin protein levels. CL: cell lysate, Exo: exosome lysate. (**C**) Western blot analysis of exosomes isolated from HEK293T WT, DNAJB6-WT, and DNAJB6-M3 for DNAJB6, GFP, and Calnexin protein levels. DNAJB6A: 40 kDa, DNAJB6B: 25 kDa, GFP-DNAJB6(-M3): 50 kDa. (**D**) Representative image of DNAJB6-WT exosome uptake in HEK293T recipient cells (t = 24 h).

In order to examine the presence of DNAJB6 in EVs, EVs were isolated from cell culture supernatants from HEK293T cells employing sequential centrifugation (Figure 2A). Western blotting analysis of the EVs and cell lysates showed that both DNAJB6 isoforms, A (40 kDa) and B (25 kDa), were present in cells, while the EV fractions from HEK293T cells contained only the shorter DNAJB6b isoform, albeit at low levels (Figure 2B). The absence of DNAJB6a and presence of DNAJB6b in EVs could be explained by the different localization of the two DNAJB6 splice forms in cells; i.e., DNAJB6a is predominantly present in the nucleus, while DNAJB6b is present in both the cytosol and nucleus [27,28,29,30]. As a quality control, we verified if the EV fractions contained higher amounts of EV-specific protein TSG101 and low levels of non-EV specific protein Calnexin (an ER marker protein) as compared to cell lysates (Figure 2B).

Next, we examined whether overexpression of DNAJB6b in producer cells was reflected in the amount of DNAJB6b in EVs. To this end, we generated GFP-DNAJB6b-WT and GFP-DNAJB6b-M3 HEK293T cell lines and isolated EVs from the respective cell culture media. Of note, GFP fusion to DNAJB6b does not have an effect on the cytoplasmic location or function of DNAJB6b [26,27,31]. Figure 1C shows that EVs isolated from overexpression cells contained high amounts of GFP-DNAJB6b(-M3), which was enriched compared to the whole cell lysate (Figure 2C). The DNAJB6b-M3 expression was lower than that of DNAJB6b-WT in HEK293T cells, which has been observed before for HEK293T cells [26] (Figure 2C). When EVs isolated from GFP-DNAJB6b-WT overexpression cells were added to recipient HEK293T cells, green punctate structures were visible in the cell cytosol, suggesting cellular uptake of EVs via endocytosis (Figure 2D). The present findings, in conjunction with reported results showing transmission of HSP40 and HSP70 via EVs14, suggest that cells may use EVs to deliver chaperones as a general mechanism to impact protein folding in target cells, and EVs loaded with DNAB6b thus hold promise as a chaperone delivery system.

### 3.2. Label-Free Mass-Spectrometry-Based Proteomics of EVs

**Figure 3:**
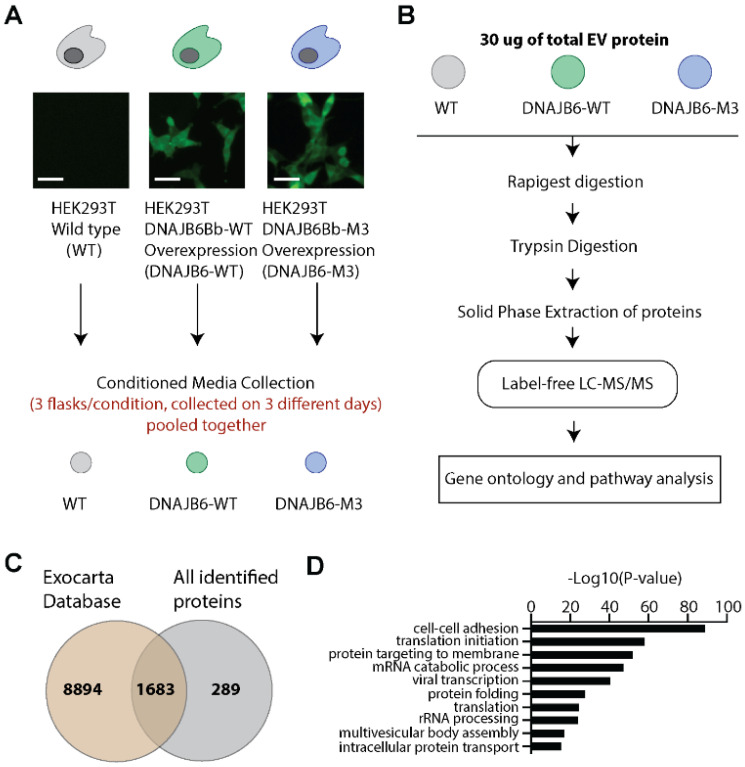
Global profiling of EV proteins identified using mass spectrometry. (**A**) Experimental setup for obtaining the WT, DNAJB6-WT, and DNAJB6-M3 EVs. (**B**) Workflow for mass-spectrometric analysis of EVs obtained in (**A**). (**C**) Comparative analysis of proteins identified in the isolated EVs to the published EV proteome in ExoCarta using Venn diagrams. (**D**) Top 10 significantly enriched GO biological processes in EVs under study.

We next employed label-free mass-spectrometry-based proteomics to characterize the protein contents of WT, DNAJB6-WT, and DNAJB6-M3 EV populations. Towards this end, we generated stable cell lines overexpressing DNAJB6-WT and DNAJB6-M3 for attaining high EV yields in a cost-effective manner. Next, we isolated EVs from the stable cell lines and the HEK293T WT cell line. (Figure 3A). The EVs were digested by Rapigest, and the proteins were trypsin-digested and subjected to LC-MS/MS analysis (Figure 3B). Using bioinformatic analysis, we identified a total of 1975 proteins (Figure 3C and Supplementary Table S1). Comparative analysis of the identified proteins with published EV proteome data compiled in ExoCarta [32] (www.exocarta.org; accessed 1 August 2019) showed a major overlap of 1683 proteins (Supplementary Table S2). This list included the common EV proteins, such as annexins, integrins, EV marker proteins such as CD81, CD9, TSG101, and Alix, heat shock proteins, and housekeeping proteins. Gene ontology (GO) biological processes that were associated with proteins enriched in the isolated EVs included cell–cell adhesion, multivesicular body assembly, protein folding, and intracellular protein transport (Figure 3D). Further, proteins (n = 289) that were exclusively present in the isolated EVs (Supplementary Table S2) were functional in biological processes underlying mitochondrial function (e.g., ALDH1B1, MT-CO2, GRPEL1, ATP5F1, AIFM1, IDH3G, etc.), endoplasmic function (e.g., SEC61G, TMTC3, ERO1A, TMEM258, NRBP1), and cytoskeleton function (e.g., GPHN, PAK1, FGD1, FMN2, CDC42SE1), which could be attributed to protein contamination from other cellular compartments carried over during isolation. Next, we determined tissue annotation using up-tissue analysis. Surprisingly, the largest fraction encompassing 56% proteins was found to be enriched in the brain despite the renal origin of HEK293T cells, which would corroborate the neuronal features of HEK293T cells [33,34] (Table 1). Importantly, no disease pathogenesis pathway was associated with the enriched proteins in the EVs, as determined by the KEGG pathway analysis (Table 2), which is important for their safe use as delivery vehicles.

### 3.3. WT, DNAJB6-WT, and DNAJB6-M3 EVs Show Common Protein Markers with Some Unique Proteins

**Figure 4:**
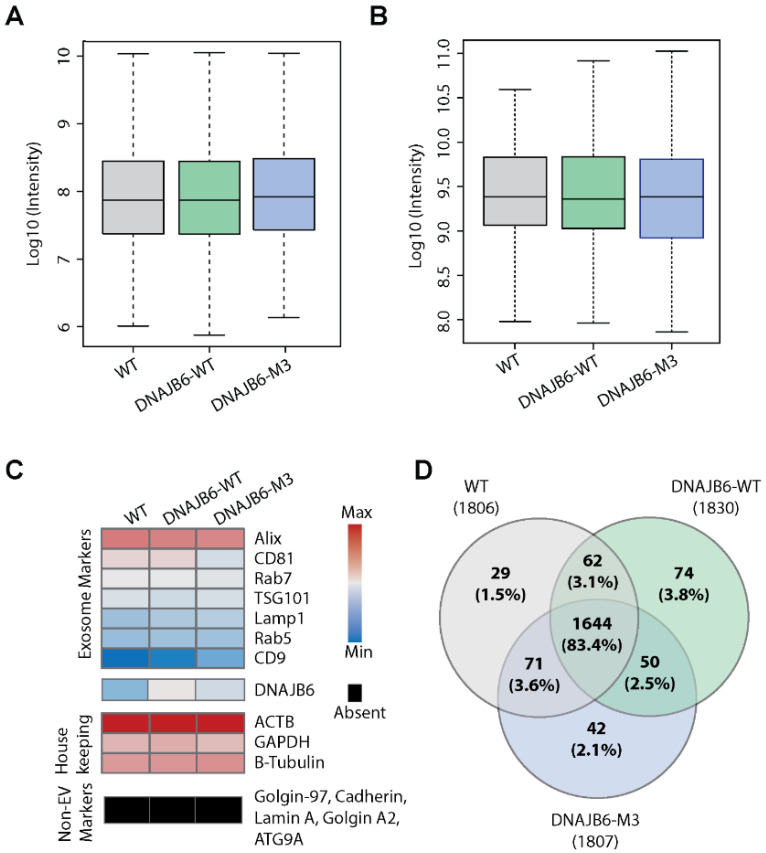
WT, DNAJB6-WT, and DNAJB6-M3 EVs show similar proteomes. (**A**) Comparative analysis based on total intensity values of protein datasets in WT, DNAJB6-WT, and DNAJB6-M3 EVs. (**B**) Comparative analysis based on intensity values of top 100 EV marker proteins from ExoCarta database. (**C**) Intensity heatmap for representative EV marker, non-EV specific proteins, housekeeping proteins, and DNAJB6. (**D**) Venn diagram of proteins found specifically in WT, DNAJB6-WT, and DNAJB6-M3 EVs.

We next set out to investigate the differences in EV proteomic profiles upon DNAJB6 overexpression. The intensity values of protein expression were highly similar amongst the EV types, therefore motivating us to investigate individual protein levels for differences (Figure 4A). This observation also suggests that the overall proteome contents across EVs remain conserved. Next, we examined the intensities of the top 100 EV marker proteins from ExoCarta. This analysis revealed that the EV marker protein profiles remained similar, confirming that the common EV protein profiles did not change dramatically across subtypes, albeit with subtle differences in intensities (Figure 4B). Importantly, non-exosome marker proteins, such as Golgi97, Lamin A, and Cadherin, were absent in all EV types (Figure 4C). DNAJB6 was present in low quantities in WT EVs and in relatively higher quantities in DNAJB6-WT and DNAJB6-M3 EVs (Figure 4C), corroborating the Western blotting analysis (Figure 2C) and thus suggesting expression-dependent loading of DNAJB6 in EVs (Figure 2C).

Finally, we compared the proteomic profiles between the three EV types for differences in protein expression. A major portion of the proteome was shared among EVs, with 1644 (83.4%) proteins in common and <4% proteins unique for each EV type (Figure 4D, Supplementary Table S3). GO term enrichment analysis of the 29 unique proteins in WT EVs (NDUFA6, NDUFS1, IDH3B, IDH3G, SUCLG1, SUCLA2, GLRX5, MT-CO3, etc.) revealed enrichment of the tricarboxylic acid cycle and mitochondrial electron transport, while the 74 proteins exclusively present in DNAJB6-WT EVs (VTI1A, VTI1B, VPS29, TRAPPC3, GAK, RAB6C, etc.) were associated with Golgi-to-vacuole and intra-Golgi vesicle transport (Supplementary Figure S2, Supplementary Table S3). Further, the 42 proteins that were uniquely present in DNAJB6-M3 EVs showed enrichment in autophagy and apoptosis. As most proteins remained approximately similar in all three EV types, this suggested that the differences between EVs were minor in terms of the biological processes they might affect in recipient cells.

### 3.4. Differentially Regulated Proteins Induced by DNAJB6 Overexpression

**Figure 5:**
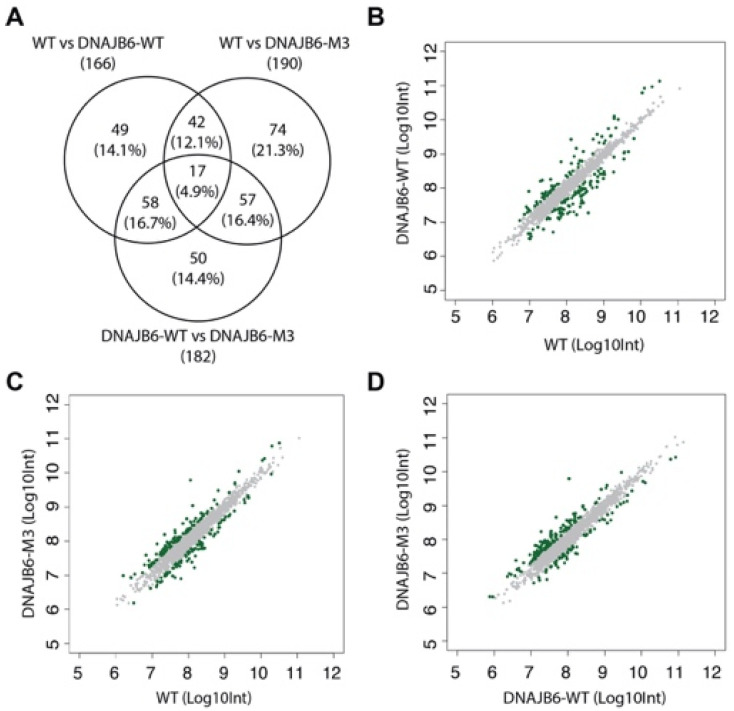
Identification of differentially expressed proteins among WT, DNAJB6-WT, and DNAJB6-M3 EVs. (**A**) Venn diagram of differentially expressed proteins in WT, DNAJB6-WT, and DNAJB6-M3 EVs. Scatter plot depicting differential intensity values for proteins found in (**B**) WT and DNAJB6-WT, (**C**) WT and DNAJB6-M3, and (**D**) DNAJB6-WT and DNAJB6-M3. Green dots represent proteins with differential expression of FC > 2, i.e., >|Antilog (0.3)|. Int: intensity value.

In order to study how proteomes of HEK293T EVs are fine-modulated upon DNAJB6 overexpression, the WT EVs were quantitatively compared with DNAJB6-WT and DNAJB6-M3 EVs. Differentially expressed proteins were selected by considering |FC| > 2. With this cutoff, 166, 190, and 182 proteins were found to be differentially expressed in comparisons between WT and DNAJB6-WT EVs, WT and DNAJB6-M3 Evs, and DNAJB6-WT and DNAJB6-M3 EVs, respectively (Figure 5A, Supplementary Table S4). DNAJB6 overexpression mediated loading of DNAJB6 as well as its predicted interacting proteins (UCSC Gene Interactions tool [35], http://genome-euro.ucsc.edu/cgi-bin/hgGeneGraph), such as TOP1, HIST2H3A, HSPB1, SLC25A3, TUFM, LONP1, and SLC25A13, in the EVs. There were also some unique protein entries that may hint at a differential effect of the DNAJB6 mutant in contrast to DNAJB6-WT on the cellular environment, which needs further experimental validation. Further, 48, 74, and 50 proteins were differentially regulated across the WT, DNAJB6-WT, and DNAJB6-M3 comparisons (Figure 5A). Among the top upregulated proteins between WT and DNAJB6-WT EVs were DNAJB6 (FC 20.6), TOP1 (FC 8.5), KRT9 (FC 8), HSPB1 (FC 2.4), and Histones (H1F0, HIST1H2AC, HIST1H1E, HIST2H3A, HIST1H1C, etc.). On the other hand, the downregulated proteins included mitochondrial proteins, such as HADHA (FC 20.5), SLC25A13 (FC 11), DARS2 (FC 8.5), and LONP1 (FC 8.19) (Figure 5B, Supplementary Table S4).

Next, the WT and DNAJB6-M3 EVs were assessed for differentially regulated proteins. As expected, DNAJB6 was present among the top differentially enriched proteins. Among possible DNAJB6 interactors, the dataset only contained HSPB1 (FC 4.9), while the rest of the proteins were different from the hits from the comparison between WT and DNAJB6-WT EVs (Figure 5C). Interestingly, JAKMIP3, a microtubule-interacting protein, was highly upregulated in EVs upon DNAJB6-WT (FC 8.3) as well as M3 (FC 4.6) overexpression (Supplementary Table S4). Recently, it was shown that DNAJB6a interacts with microtubules and affects spindle assembly during mitosis [36]. Whether overexpression of DNAJB6b may lead to its unusual interaction with microtubules remains to be investigated.

Finally, to understand whether mutations in the S/T rich segment in DNAJB6-WT could lead to differential loading of DNAJB6 and other proteins into EVs, the DNAJB6-WT and DNAJB6-M3 EV protein profiles were compared (Figure 5D, Supplementary Table S4). Ribosomal proteins, histones, and cell adhesion proteins were upregulated in the DNAJB6-M3 EVs compared to the DNAJB6-WT EVs. Interestingly, NDRG2 protein was highly upregulated in DNAJB6-M3 EVs (FC 58). NDRG2 is a tumor suppressor gene in various tumors and has a function in cell proliferation, differentiation, and apoptosis [37]. It is possible that overexpression of DNAJB6-M3 and subsequent deregulated proteostasis due to low chaperone activity may lead to tumorigenesis. NDRG2 may then be upregulated in response to suppress this phenotype. Whether there is a causal relationship between NDRG2 upregulation and DNAJB6 downregulation requires experimental verification. In summary, DNAJB6 upregulation led to differential upregulation of predicted DNAJB6 interacting proteins and some heat shock proteins, which suggests that this strategy, when used for therapeutic intervention, may have a synergistic favorable effect by protein partners of DNAJB6.

### 3.5. Overview of the Protein Folding Environment in WT, DNAJB6-WT, and DNAJB6-M3 EVs

**Figure 6:**
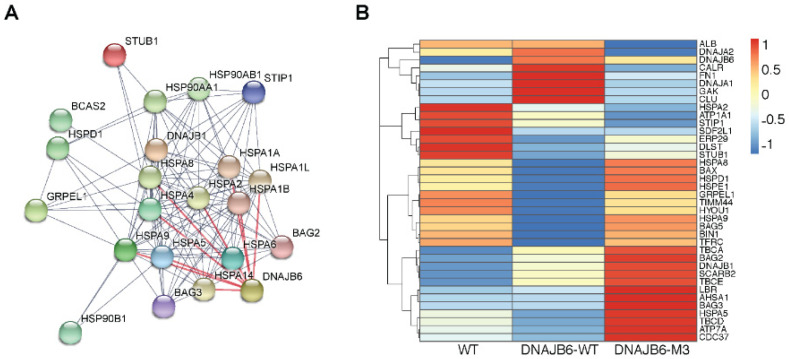
Comparative analysis of chaperones among WT, DNAJB6-WT, and DNAJB6-M3 EVs. (**A**) Predicted interacting partners of DNAJB6 and their interactions using STRING database. (**B**) Heatmap of row-scaled intensity values of proteins involved in protein folding found in WT, DNAJB6-WT, and DNAJB6-M3 EVs.

Our data pointed out that some heat shock proteins and DNAJB6 interacting proteins were upregulated in EVs produced by DNAJB6b-overexpressing HEK293T cells. Regarding careful analysis of this enrichment, we first identified predicted interacting partners of DNAJB6 in the protein folding protein set in the STRING database (https://version-11-0.string-db.org/) [38] (Figure 6A). We next compared intensity changes of direct and indirect interaction partners of DNAJB6 across EV types (Figure 6B). Along with DNAJB6, DNAJA1 (HSP40 A1), DNAJA2 (HSP40 A2), FN1 (Fibronectin 1), CALR (Calreticulin), GAK (Cyclin G Associated Kinase), and CLU (Clusterin) were enriched in EVs upon DNAJB6-WT overexpression with respect to WT and DNAJB6-M3 Evs. Interestingly, some proteins were present in lesser quantities in DNAJB6-WT Evs with respect to both WT and DNAJB6-M3 Evs, i.e., HSPA8, BAX, HSPD1, HSPE1, GRPEL1, TIMM44, HYOU1, HSPA9, BAG5, BIN1, and TFRC. Last, some proteins were present in higher quantities in DNAJB6-M3 EVs as compared to WT and DNAJB6-WT EVs. For example, TBCA, BAG2, DNAJB1, SCARB2, TBCE, and LBR among others belonged to this group of proteins. It can be speculated that DNAJB6 overexpression in EV-producer cells positively impacts the EV-contained protein folding chaperone machinery by inducing co-loading of other chaperones. Thus, EVs loaded with DNAJB6 by DNAJB6 overexpression in producer cells may hold potential as therapeutic delivery vehicles and might lead to synergistic effects coming from other chaperones co-loaded along with DNAJB6. Further, it is worthwhile to note that some chaperone clusters were downregulated upon overexpression of DNAJB6b, which may suggest the presence of compensatory mechanisms.

## 4. Discussion and Conclusions

Current research points towards a possible role of EV-mediated chaperone transmission as a clinically potent restoration mechanism for a disturbed cellular protein environment [14,15], typical of neurodegenerative disorders such as Huntington’s and Alzheimer’s disease [16]. Underlying the realization of the therapeutic potential of the aforementioned mechanism, DNAJB6, being among the most effective modulators of protein folding [26], advances as the most promising candidate regarding treatments for the brain. Interestingly, EV-mediated chaperone delivery was suggested to contribute to the cross-cellular restoration of proteostasis by astrocytic overexpression of DNAJB6 [15]. The present study corroborated this earlier report and, in addition to that, provided an in-depth molecular characterization of the protein cargo in DNAJB6b-enriched EVs, which is considered pivotal for the progress of EVs towards clinical use. We first showed that cells overexpressing DNAJB6-WT are able to reduce polyQ aggregation in recipient cells in a co-culture system that precludes direct cell–cell contact. Next, EVs isolated from DNAJB6-WT-overexpressing cells were shown to contain a high amount of DNAJB6, suggesting EV-mediated chaperone transfer from DNAJB6-overexpressing cells to the recipient cells.

When introducing therapeutic cargo in EVs through endogenous loading, e.g., by overexpression of the therapeutic protein in EV producer cells, it is important to not only confirm the presence of the protein of interest but also to analyze the complete EV protein content. In addition to the protein of interest, interacting proteins are likely to become co-loaded into the EVs and may affect EV function in recipient cells [39,40,41,42,43]. In the present study, we employed proteomic characterization of EVs isolated from WT, DNAJB6-WT, and DNAJB6-M3 overexpression cell lines and observed that EV proteins were not associated with disease pathogenesis pathways, as determined by the GO pathway analysis with OMIM disease database.

Interestingly, DNAJB6-WT overexpression in cells led to loading of DNAJB6, as well as other related chaperone proteins, in EVs. For example, DNAJA1, DNAJA2, and HSP90 were present in greater quantities in DNAJB6-WT EVs as compared to WT EVs, suggesting a possible additive effect towards proteostasis improvement. Additionally, they may inhibit oxidative stress [44] and alleviate autoimmune responses, e.g., in arthritis [45]. However, while DNAJB6-M3, a mutant form of DNAJB6, overexpression led to reduced loading of chaperone proteins in EVs, a significant downregulation of another set of proteins outside the DNAJB6-interactome, e.g., STUB1, DLST, SDF2L1, and GAK, was also observed. This warrants further study to characterize the underlying biological processes.

Because cellular stress leads to release of EVs that contain high amounts of chaperones [14], proteomic characterization of EVs could be employed to detect a disturbed protein folding environment of cells in order to diagnose disease. Moreover, given the early appearance of disease-causing proteins, such as alpha-synuclein, amyloid precursor protein, and tau in EVs, together with the presence of these EVs in the blood [46], EVs represent an opportunity for early detection of disease in a minimally invasive manner.

Collectively, our data present the comprehensive examination of EVs derived from HEK293T cells under normal and DNAJB6 overexpression conditions and indicate that comparative proteomics can allow extraction of useful and novel information about EVs, reflecting their biological status and fitness as drug delivery systems.

## Figures and Tables

**Table 1:** Up-tissue analysis for proteins enriched in WT, DNAJB6-WT, and DNAJB6-M3 EVs: top 10 tissues are listed.

Term	Gene Count	% of Total Genes	−log10 (*p*-Value)
Brain	1051	53.8	23.3372422
Placenta	586	30	37.7695511
Epithelium	582	29.8	72.79588
Liver	428	21.9	42.3767507
Skin	339	17.4	23.6197888
Platelet	245	12.6	92.2839967
Muscle	201	10.3	25.9586073
Fetal brain cortex	144	7.4	69.3187588
Cajal–Retzius cell	140	7.2	77.2218487
B-cell lymphoma	84	4.3	37.1674911

**Table 2:** Top 10 KEGG pathways for proteins in WT, DNAJB-WT, and DNAJB6-M3 EVs.

Term	Gene Count	−log10 (*p*-Value)
WT EVs		
Endocytosis	99	21.7692843
Ribosome	69	21.1538224
Biosynthesis of antibiotics	81	15.41937
Carbon metabolism	49	11.6209114
Adherens junction	36	10.8601157
Biosynthesis of amino acids	35	9.91354735
Protein processing in endoplasmic reticulum	60	9.87526371
Regulation of actin cytoskeleton	69	9.63444588
Citrate cycle (TCA cycle)	21	9.577817
Focal adhesion	67	9.14623656
DNAJB6-WT EVs		
Endocytosis	103	24.1796338
Ribosome	67	19.5762603
Focal adhesion	73	11.9117448
Regulation of actin cytoskeleton	73	11.4463798
Adherens junction	36	10.8238366
Biosynthesis of antibiotics	72	10.7452565
Protein processing in endoplasmic reticulum	61	10.3254685
Proteoglycans in cancer	65	8.80851114
Shigellosis	31	8.68976131
Pathogenic Escherichia coli infection	27	8.59323546
DNAJB6-M3 EVs		
Endocytosis	101	22.9111633
Ribosome	67	19.5762603
Biosynthesis of antibiotics	78	13.7349218
Adherens junction	37	11.5832988
Regulation of actin cytoskeleton	72	10.965138
Protein processing in endoplasmic reticulum	62	10.8381002
Focal adhesion	68	9.53814583
Carbon metabolism	45	9.174472
Biosynthesis of amino acids	33	8.50374616
Pathogenic Escherichia coli infection	26	7.83547924

## Data Availability

The datasets are provided in XLSX file in the Supplementary Materials.

## Supplementary Materials

The following supporting information can be downloaded at: https://www.mdpi.com/article/10.3390/pharmaceutics14112485/s1, Figure S1: DNAJB6 does not contain putative secretion signal peptide; Figure S2: Top 10 GO biological processes enriched for proteins exclusively present in WT, DNAJB6WT, and DNAJB6-M3 EV; Table S1: Total proteins present in WT, DNAJB6WT, and DNAJB6-M3 EVs; Table S2: Proteins overlapping with ExoCarta database; Table S3: Proteins overlapping in WT, DNAJB6WT, and DNAJB6-M3 EVs; Table S4: Proteins differentially expressed in comparisons between WT and DNAJB6-WT EVs, WT and DNAJB6-M3 EVs, DNAJB6-WT and DNAJB6-M3 EVs.
