# Supplementary material for: DNAJB6-Containing Extracellular Vesicles as Chaperone Delivery Systems: A Proteomic Analysis
Source: Pharmaceutics. 2022 Nov 17;14(11):2485. doi: 10.3390/pharmaceutics14112485 (PMC9698516; doi:10.3390/pharmaceutics14112485)
Supplement: Supplementary file 1 [file pharmaceutics-14-02485-s001.zip › pharmaceutics-1975482-supplementary.pdf]

pharmaceutics

# Supplementary Information

# DNAJB6- containing Extracellular Vesicles as Chaperone Delivery Systems: a proteomic analysis

**Bhagyashree S. Joshi [1], Hector Garcia Romeu [2], Aldy Aliyandi [2], Marcel P. de Vries [3] and Inge S. Zuhorn [1,*]**

[1] Department of Biomedical Engineering, University Medical Center Groningen, University of Groningen, Antonius Deusinglaan 1, 9713AV Groningen, The Netherlands
[2] Groningen Research Institute of Pharmacy, University Medical Center Groningen, University of Groningen, Antonius Deusinglaan 1, 9713AV Groningen, The Netherlands
[3] Interfaculty Mass Spectrometry Center, University Medical Center Groningen, University of Groningen, Antonius Deusinglaan 1, 9713AV Groningen, The Netherlands
* Correspondence: i.zuhorn@umcg.nl

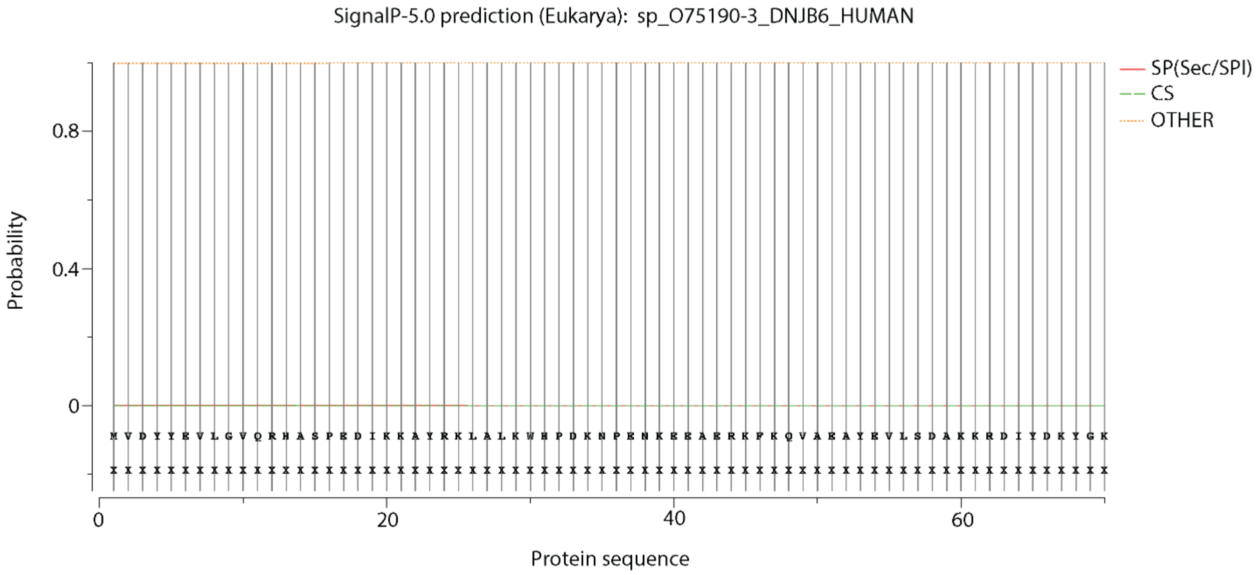


| Protein type | Signal Peptide (Sec/SPI) | Other |
|---|---|---|
| Likelihood | 0.0009 | 0.9991 |

**Figure S1**. DNAJB6 does not contain putative secretion signal peptide. (A) Secretion signal peptide prediction using SignalP5.0 for Human DNAJB6 protein (Uniprot ID: O75190). SP: Signal peptide, CS: Cleavage Site.

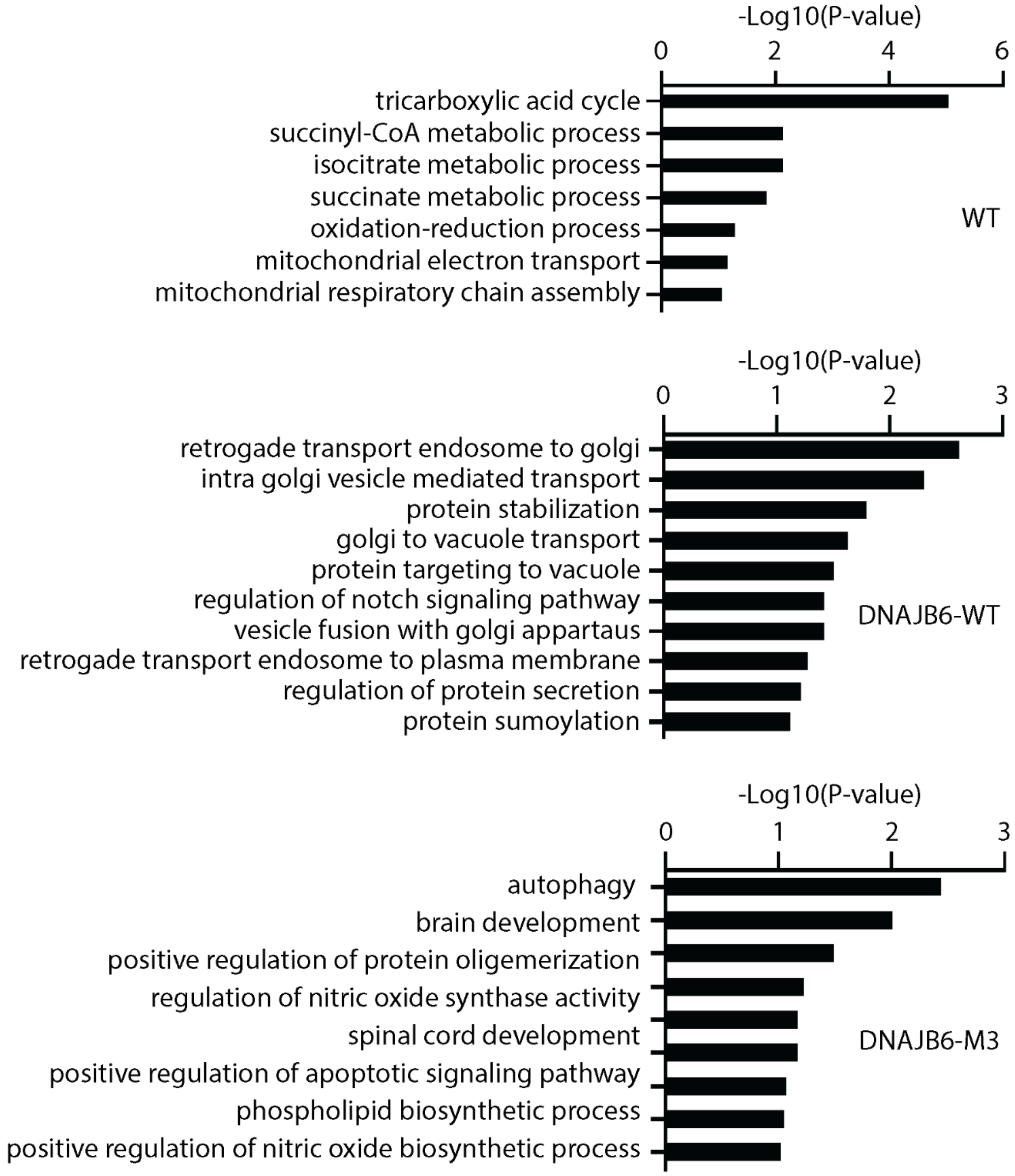


**Figure S2**. Top 10 GO biological processes enriched for proteins exclusively present in WT, DNAJB6WT and DNAJB6-M3 EV.

**Table S1**. Total proteins present in WT, DNAJB6WT and DNAJB6-M3 Evs.
https://docs.google.com/spreadsheets/d/1uKpiwEXgnSYelxAt7qAnI3HDb_x8YG9VnGz8TXJ4O6E/edit?usp=sharing

**Table S2**. Proteins overlapping with ExoCarta database.
https://docs.google.com/spreadsheets/d/1PZCFGSxoF8b3GE6a_F8VFZN_fAEEGiE60ocl_m68YEM/edit?usp=sharing

**Table S3**. Proteins overlapping in WT, DNAJB6WT and DNAJB6-M3 EVs.
https://docs.google.com/spreadsheets/d/1aIPQ29H5BEMNSCofb0edjddLdv3nMWevRVd1z8jMPsI/edit?usp=sharing

**Table S4**. Proteins differentially expressed in comparisons between WT and DNAJB6-WT EVs, WT and DNAJB6-M3 EVs, DNAJB6-WT and DNAJB6-M3 EVs.
https://docs.google.com/spreadsheets/d/1QH2bUDGLb8dJBdt6MLFpgn8tb8XhfASSymEah1Eikuc/edit?usp=sharing
